# Sonoplasma Technology for Water Treatment Against Phytopathogenic Fungi: Responses of Melanized and Hyaline Species

**DOI:** 10.3390/jof12070487

**Published:** 2026-07-02

**Authors:** Elena V. Fedoseeva, Yulia D. Sergeeva, Svetlana V. Patsaeva, Anna V. Kamler, Egor S. Mikhalev, Anna M. Lazareva, Vera A. Terekhova

**Affiliations:** 1 A.N. Severtsov Institute of Ecology and Evolution, 119071 Moscow, Russia; sergeeva.yulia.dm@gmail.com; 2 Faculty of Soil Science, M.V. Lomonosov Moscow State University, 119991 Moscow, Russia; vterekhova@gmail.com; 3 Faculty of Physics, M.V. Lomonosov Moscow State University, 119991 Moscow, Russia; spatsaeva@mail.ru; 4 N.S. Kurnakov Institute of General and Inorganic Chemistry RAS, 119071 Moscow, Russia; abramova@physics.msu.ru (A.V.K.); mikhalevec@gmail.com (E.S.M.); 5 Institute for African Studies RAS, 123001 Moscow, Russia; lazarevaam@my.msu.ru

**Keywords:** sonoplasma technology, advanced oxidation processes, hydrogen peroxide, fungal contamination, filamentous fungi, hormesis, melanin

## Abstract

Sonoplasma treatment (SPT), which combines hydrodynamic cavitation with low-temperature plasma discharge in water, has been proposed as an advanced oxidation process for reducing biological contamination. By generating physical stressors and reactive oxygen species, including hydrogen peroxide (HP), SPT may inactivate microorganisms, but its effects on stress-resistant filamentous fungi remain insufficiently characterized. We examined two phytopathogenic fungi with contrasting pigmentation: melanized *Alternaria alternata* and hyaline *Fusarium solani*. Spore suspensions were exposed to direct and indirect SPT at 30 kHz, and viability, biomass accumulation, conidial production, allelopathic activity, and pigmentation-associated spectral responses were assessed immediately after treatment and after storage. Fungus *F. solani* showed greater susceptibility, with reduced colony-forming capacity and suppressed biomass production, although surviving propagules showed increased sporulation. In contrast, *A. alternata* maintained viable growth under the tested conditions and showed stimulation of growth-related and reproductive endpoints, together with darker colony pigmentation. These responses are consistent with pigmentation-associated tolerance to SPT-induced physical and oxidative stress, but do not establish melanin as the sole causal mechanism. SPT efficacy against filamentous fungi is therefore species-dependent and may be limited when resistant melanized taxa are present.

## 1. Introduction

Biological pollution of water, characterized by the contamination of aquatic ecosystems and potable water with pathogens and other microorganisms, such as viruses, bacteria, fungi, and protozoa, represents a persistent threat to public and ecosystem health [1,2,3]. Among these contaminants, fungi are particularly difficult to control and disinfect, a challenge attributed to their complex cell structure and persistent life cycle stages [3]. In water systems, fungi are present as both dispersed spores and as mycelial aggregates. These hyphal aggregates form the structural core of biofilms, a mode of growth that significantly enhances their tolerance to environmental stresses [4]. The capacity to assume these resilient forms, including both stress-resistant spores and complex biofilms, poses a particular problem for disinfection, as both life stages have demonstrated pronounced resilience to chemical and thermal shocks [5]. In aquatic environments, mycelial fungi from the division Ascomycota are predominant. Frequently isolated genera include *Aspergillus*, *Alternaria*, *Penicillium*, *Trichoderma*, *Cladosporium*, and *Fusarium*, many of which contain species of significant phytopathological and public health concern [6,7,8,9].

Conventional strategies for managing fungal contamination encompass both chemical and physical methods [3]. Chemical disinfection typically relies on powerful oxidizing agents including chlorine-based compounds, peracetic acid, ozone, and hydrogen peroxide (HP) [6,7,9,10,11]. Many chemical-disinfection studies report greater resistance among melanized fungi, with *Aspergillus niger* often identified as the most tolerant species [9,11].

Similarly, physical methods, such as ultraviolet (UV) irradiation, one of the most common non-chemical approaches [6,8,12,13,14], also reveal a wide spectrum of sensitivity across fungal genera. While UV inactivation is effective against a broad range of fungi, including *Aspergillus*, *Penicillium*, *Trichoderma*, *Fusarium*, *Cladosporium*, and *Trichophyton*, resistance levels vary significantly. Although it is difficult to rank species by resistance due to disparate literature data, a recurring pattern emerges: non-melanized species, such as *F. solani*, are consistently among the least resistant [6], whereas melanized fungi, such as *Cladosporium* sp., exhibit much higher survival rates [8]. This suggests that intrinsic cellular traits, rather than the specific inactivation method, are important determinants of survival. This observation extends even to mechanical disinfection strategies, such as those employing ultrasound or shock waves [15,16], where the structural and chemical resilience of the fungal cell wall continues to play a critical role in resistance.

In response to the limitations and variable efficacy of conventional methods, advanced oxidation processes (AOPs) have been developed as promising alternatives. These technologies are defined by their ability to generate powerful, non-selective oxidants, primarily reactive oxygen species (ROS) [17]. Classic disinfection methods, such as UV irradiation, are frequently integrated into AOPs. This integration occurs either by combining UV with chemical species (e.g., the UV/H_2_O_2_ system) or by implementing it under specialized conditions that augment ROS generation. For instance, a recent study investigated the fungicidal spectrum of phenolic-acid derivatives (including cinnamic and benzoic acids) within a photocombination system using 405 nm violet light. This combined method demonstrated high efficiency for the disinfection of pathogenic and spoilage fungi [18].

A recent development In this field Is sonoplasma treatment (SPT), a combined AOP that integrates hydrodynamic cavitation with low-temperature plasma discharge directly within the aqueous medium [19,20,21]. This synergistic combination produces a multicomponent stress environment characterized by localized extreme temperatures, powerful shock waves, an electromagnetic field, and, notably, the dissociation of water molecules. This dissociation leads to the prolific formation of ROS, including hydroxyl radicals (•OH), HP (H_2_O_2_), and ozone (O_3_) [20,22]. The efficacy of SPT has been demonstrated against a range of organic pollutants (e.g., methanol, antibiotics, and dyes) and biological contaminants (bacterial cells *Escherichia coli*, fungal cells *Saccharomyces cerevisiae*, and bacterial-fungal complex of microorganisms in irrigation waters) [20,21,23,24,25]. Its mode of action is thought to involve both physical disruption of cell membranes by cavitation and shock waves and oxidative damage caused by ROS. These ROS can damage cellular organelles, such as mitochondria, and interfere with critical metabolic systems, including the catabolic pathways responsible for adenosine triphosphate (ATP) synthesis [18].

Although SPT demonstrates high efficacy against bacteria such as *E. coli* and the yeast *S. cerevisiae* [24], its interaction with filamentous fungi appears considerably more complex. Laboratory studies show that SPT applied to bacterial-fungal complexes in greenhouse irrigation waters exhibits substantially reduced efficacy against filamentous fungi compared to bacteria [25]. The inhibitory effect on microscopic fungal propagules is directly proportional to the treatment regime. Among the filamentous fungal colonies that grew, a high proportion were hyaline mycelial forms, which include most phytopathogenic species of the genus *Fusarium*. Colonies of *Penicillium* spp., *Trichoderma* spp., *Cladosporium* spp. And *Alternaria* spp. Were less frequent.

This fungal resistance has been associated with the complex architecture of the cell wall, which contains chitin and, in many taxa, the phenolic pigment melanin. Melanin plays vital physiological and ecological roles, conferring broad-spectrum resistance by scavenging ROS, mitigating UV-induced damage, and enhancing both structural integrity and virulence in melanized forms [26,27,28,29,30]. However, despite the well-established protective role of melanin against diverse stressors, a significant knowledge gap remains: how pigmentation-associated traits modulate the physiological response of filamentous fungi to the combined stressors imposed by SPT remains insufficiently characterized.

One relevant framework for interpreting such responses is hormesis, a dose–response relationship in which low levels of a toxic or physical stressor trigger stimulatory effects, while high doses lead to cellular inhibition or death [31,32]. This phenomenon has also been documented in fungi. For instance, HP has been shown to induce hormesis in yeasts such as *S. cerevisiae* [33]. Nevertheless, the relationship between pigmentation-associated tolerance and post-treatment physiological recovery remains unexplored. Specifically, it is not yet understood whether pigmentation-associated traits may buffer SPT-induced oxidative stress sufficiently to shift the response from inactivation toward recovery, stimulation, or enhanced reproductive output.

On this basis, we used a two-species comparative model to examine whether contrasting pigmentation phenotypes are associated with different physiological responses to sonoplasma-induced stress. We compared two phytopathogenic fungi with broadly similar growth characteristics but distinct pigmentation traits: the melanized *Alternaria alternata* and the hyaline *Fusarium solani*. Rather than testing melanin causality directly, this design was intended to evaluate how pigmentation-associated traits may influence fungal survival, growth recovery, sporulation, dissolved organic compound profiles, allelopathic activity, and pigmentation responses after SPT. These endpoints were assessed immediately after treatment and following storage to distinguish immediate inactivation from persistent or delayed physiological effects. This approach allowed us to evaluate the antifungal efficacy and potential selectivity of SPT under controlled laboratory conditions and to identify whether resistant fungal phenotypes may require optimized or complementary treatment strategies in water sanitation.

## 2. Materials and Methods

### 2.1. Fungal Strains and Cultivation Conditions

Two fungal strains, isolated from soil, were selected as models for their contrasting pigmentation phenotypes and cell wall properties: the heavily melanized *Alternaria alternata* (Fr.) Keissl (GenBank accession number BankIt3043361 513 PX883736) and the hyaline (non-melanized) *Fusarium solani* (Mart.) Sacc., 1881 (GenBank accession number BankIt3043361 515 PX883737). Both are facultative phytopathogens of significant agricultural concern. *A. alternata* synthesizes 1,8-dihydroxynaphthalene (DHN)-melanin, a key pigment known to confer environmental resilience in Ascomycota [34]. *F. solani* is a widespread pathogen of many agriculturally important crops [35]. This pairing was used as a comparative model to examine whether contrasting pigmentation phenotypes are associated with different responses to SPT, rather than as a direct test of melanin causality.

Fungi were cultivated on standard Czapek-Dox agar medium to promote sporulation. Spore suspensions were prepared by washing sporulating colonies with sterile distilled water. The resulting suspensions were adjusted to final densities of 10^2^ and 10^4^ conidia/mL to assess potential density-dependent treatment effects.

### 2.2. Design of Sonoplasma Treatment

SPT was applied to aqueous spore suspensions using a sonoplasma setup that combines hydrodynamic cavitation with low-temperature plasma discharge, as previously detailed [20]. Briefly, the system generates a cavitation field through which the liquid flows. Application of high-voltage pulses at an ultrasonic frequency of 30 kHz to the reactor electrodes induces plasma discharge within the cavitation bubbles. These bubbles act as microscopic reactors, generating intense physical and chemical conditions that inactivate microorganisms and degrade contaminants.

Sonoplasma treatment was carried out using a flow-through system with the following parameters:

Flow rate: 12 L/min. This parameter ensured consistent water contact with the sonoplasma discharge zone for generation of reactive oxygen species.

Sample volume per cycle: at least 5–7 L. This volume allowed for a representative sample for subsequent analysis of microbiological and physicochemical parameters.

Temperature change: 1–3 °C. The observed temperature increase was a side effect of the energy release in the cavitation zone and plasma discharge. The magnitude of the change was directly related to the power of the treatment: at maximum settings, a temperature increase of 3 °C was recorded, while at minimum settings, a temperature increase of 1 °C was observed.

The generation of reactive oxygen species (ROS) was confirmed by quantifying the concentration of HP, a stable ROS product, in the treated water immediately after processing. HP concentration was determined by permanganometric titration [36,37].

For a comprehensive analysis of the effectiveness of SPT, three experimental treatment groups were used: (1) direct SPT, in which fungal propagules were exposed directly to the plasma discharge; (2) indirect SPT, in which fungal propagules were exposed to sonoplasma-activated water after treatment; and (3) HP exposure, in which fungal propagules were exposed to exogenous HP at concentrations comparable to those formed during SPT, used as a measurable oxidative-stress reference (Table 1).

### 2.3. Effect of Direct Sonoplasma Treatment on Fungal Viability and Growth at Different Intervals After Treatment

Spore suspensions of *A. alternata* and *F. solani* were subjected to two treatment modes: double (2×) and fourfold (4×) passes through the sonoplasma reactor at 30 kHz. An untreated sample served as the control.

To evaluate the immediate and persistent effects of SPT on fungal viability and growth, treated and untreated spore suspensions were inoculated onto both solid and liquid media.

For assessment on solid media, aliquots (0.1 or 0.2 mL) from each sample were spread onto Petri dishes containing Czapek-Dox agar amended with antibiotics to suppress bacterial growth. The inoculated plates were incubated at 23–24 °C.

For assessment in liquid culture, untreated and SPT-treated spore suspensions were supplemented with mineral salts and sucrose (added as aliquots of stock solutions) to adjust the nutrient composition of the Czapek-Dox medium. One hundred-milliliter portions of these suspensions were dispensed into 250 mL flasks and incubated on an orbital shaker (250–300 rpm) at 23–24 °C to promote aerobic growth.

Viability assessments were conducted at three time points: immediately after SPT (day 0), and after 10 and 30 days of storage of the spore suspensions at 4 °C, to determine the persistence of the treatment’s effects. These storage intervals were included to reflect realistic operational scenarios in which treated irrigation water or fungal propagule suspensions may be stored before use, and to assess whether treatment effects persist over agronomically relevant timescales.

### 2.4. Quantification of Fungal Viability, Growth, and Reproduction

Fungal response to SPT was assessed using three primary endpoints: colony-forming units (CFU) and conidial productivity of grown fungal colonies on solid media, and mycelial biomass in liquid media, in both treated and untreated control samples.

The number of viable fungal propagules was determined by measuring CFU. Treated and control suspensions were serially diluted and plated onto Czapek-Dox agar medium in triplicate. Colonies were counted after 4 days of incubation.

The number of CFU in the initial inoculum was calculated using the following equation:

N = (M × R)/V,

where N is the number of CFU in 1 mL of the initial inoculum (CFU/mL); M is the average number of colonies counted on the Petri dish; R is the reciprocal of the dilution factor; and V is the volume of inoculum plated (mL).

Conidial productivity (sporulation rate) was calculated on the 10^th^ day of colony growth on solid medium and expressed as the number of conidia produced per unit area (cm^2^). Three circular sections of sporulating mycelium, extending from the colony center to the edge, were excised using a sterile microbiological corer. The sections were transferred to 5 mL of sterile distilled water containing a small amount of surfactant (Tween 80). The suspension was thoroughly mixed and filtered through a fine nylon sieve to separate conidia from hyphal fragments. The conidia concentration was quantified using a hemocytometer according to published methodological guidelines [38]:

I = (L × N)/(S × V),

where I is conidial productivity (conidia/cm^2^); L is the volume of wash water (cm^3^); N is the average number of conidia counted in the hemocytometer chamber; S is the total area of the excised agar sections (cm^2^); and V is the volume of the hemocytometer chamber used for counting (cm^3^).

Mycelial biomass was determined from liquid cultures. Treated and untreated spores were inoculated into Czapek-Dox liquid medium and cultivated for 7 days at 23–24 °C. The mycelium was then harvested by filtration onto pre-weighed, ash-free filter paper, dried at 103 °C to a constant weight, and the dry biomass was recorded (g).

### 2.5. Effect of Sonoplasma-Activated Water on the Vital Activity of Fungi at Different Intervals After Activation

To assess the indirect effects of SPT-treated water, water was first subjected to fourfold SPT at 30 kHz and then used to prepare fungal spore suspensions either 2 h after treatment (day 0) or after 10 and 30 days of water storage. An untreated water sample served as the control. The 10- and 30-day storage intervals were used to evaluate whether sonoplasma-activated water retained biologically active properties over storage periods relevant to practical water-use scenarios. This design was used to evaluate whether sonoplasma-activated water retained biologically active properties after treatment, independently of direct exposure of fungal propagules to the discharge zone. Fungal propagules were exposed to water without the addition of mineral or organic nutrient sources for 3 days. After this exposure, they were plated on agar nutrient medium to determine CFU and conidial productivity, as described in Section 2.4. Parallel suspensions were incubated for 7 days for biomass determination, as described in Section 2.4.

### 2.6. Effect of Hydrogen Peroxide on the Vital Activity of Fungi

The effect of HP at concentrations equivalent to those formed during SPT was assessed on the growth characteristics of *A. alternata* and *F. solani*. This experiment was designed as a comparative oxidative-stress assay using a measurable SPT-derived oxidant, rather than as a full mechanistic control reproducing all physical and chemical conditions generated during direct SPT. By adding the required volume of 3% HP to Czapek-Dox liquid medium, final HP concentrations of 2 and 6 mmol/L were obtained. Aliquots of spore suspensions were added to the prepared medium to adjust the final densities to 10^4^ conidia/mL. After 12 h of cultivation in the medium with HP, the spores were inoculated on Czapek-Dox agar medium. Mycelial biomass was determined after 7 days of cultivation in liquid medium. The number of CFU was determined after 4 days of cultivation on agar medium. Conidial productivity was calculated on the 10^th^ day of colony growth on agar medium.

### 2.7. Analysis of Allelopathic Activity

The effect of SPT on the production of fungal metabolites with allelopathic (growth-inhibitory) potential was assessed using a laboratory phytotest with oat (*Avena sativa* L.) as a model test-plant, following a modified method [39].

After a specified incubation period, agar plates with grown fungal colonies from SPT-treated and untreated (control) spores were used. To assess the effect of volatile or airborne fungal metabolites, the lid of each Petri dish was replaced with a new one, and twenty surface-sterilized oat seeds were placed on the inner surface of the lid, positioned above the fungal colony. The assemblies were then incubated for 96 h at 25 °C.

Following the incubation period, the germination rate and the length of roots and coleoptiles were measured for all oat seedlings. The allelopathic activity coefficient (Ca), which quantifies the inhibition of growth, was calculated for both roots and coleoptiles using the formula:

Ca = 1 – Lt/Lc,

where Ca is the coefficient of allelopathic activity; Lt is the average root or coleoptile length in the treatment group; and Lc is the average root or coleoptile length in the control group (mm).

The allelopathic impact of metabolites produced by fungi from SPT-treated spores was compared directly to that of metabolites from untreated spores.

### 2.8. Spectroscopic Analysis of Dissolved Fungal Metabolites and Growth Assessment

Changes in dissolved fungal metabolites and culture development were monitored using UV-Visible (UV-Vis) absorption and fluorescence spectroscopy. Fungal biomass was harvested after 7 days of cultivation. To analyze the dissolved metabolite fraction, samples were centrifuged and the supernatant was filtered through 0.22-μm nylon syringe filters to remove mycelial fragments and spores.

Absorption spectra of the filtered supernatants were recorded from 200 to 800 nm at 1 nm intervals using a Solar PB2201 spectrophotometer (Minsk, Belarus) with a 1 cm path length quartz cuvette. Distilled water served as the reference blank. The absorbance values at specific wavelengths were used as spectral indicators of dissolved organic compounds and culture development.

Filtered samples were diluted 20-fold with distilled water to prevent inner-filter effects. Fluorescence emission spectra at selected excitation wavelengths were measured using a CM2203 luminescence spectrometer (Minsk, Belarus).

### 2.9. Statistical Analysis

All experiments were performed with three to five independent biological replicates. Results are presented as the mean ± standard deviation (SD), with error bars on all figures representing the calculated SD.

The significance of differences between experimental variants was determined using a one-way analysis of variance (ANOVA), followed by Tukey’s honest significant difference (HSD) post hoc test for pairwise multiple comparisons. A difference was considered statistically significant at *p* < 0.05.

All statistical calculations were performed using the R statistical programming environment in conjunction with Microsoft Excel and the ExcelStat add-in.

## 3. Results

### 3.1. Chemical Parameters of the Aquatic System

SPT caused slight alkalization of the aqueous medium and increased the concentration of HP, a stable ROS product formed during treatment. The HP concentration increased with treatment intensity and reached 2.70 ± 0.05 mmol/L under the fourfold treatment mode (Table 2).

The HP concentration gradually declined during post-treatment storage. After double treatment, the HP concentration decreased to 0.50 ± 0.05 mmol/L by day 15 and to 0.40 ± 0.05 mmol/L by day 30.

### 3.2. Fungal Growth from Freshly Treated Spore Suspensions

Growth endpoints showed species-specific responses to SPT in the two model fungi.

**Figure 1:**
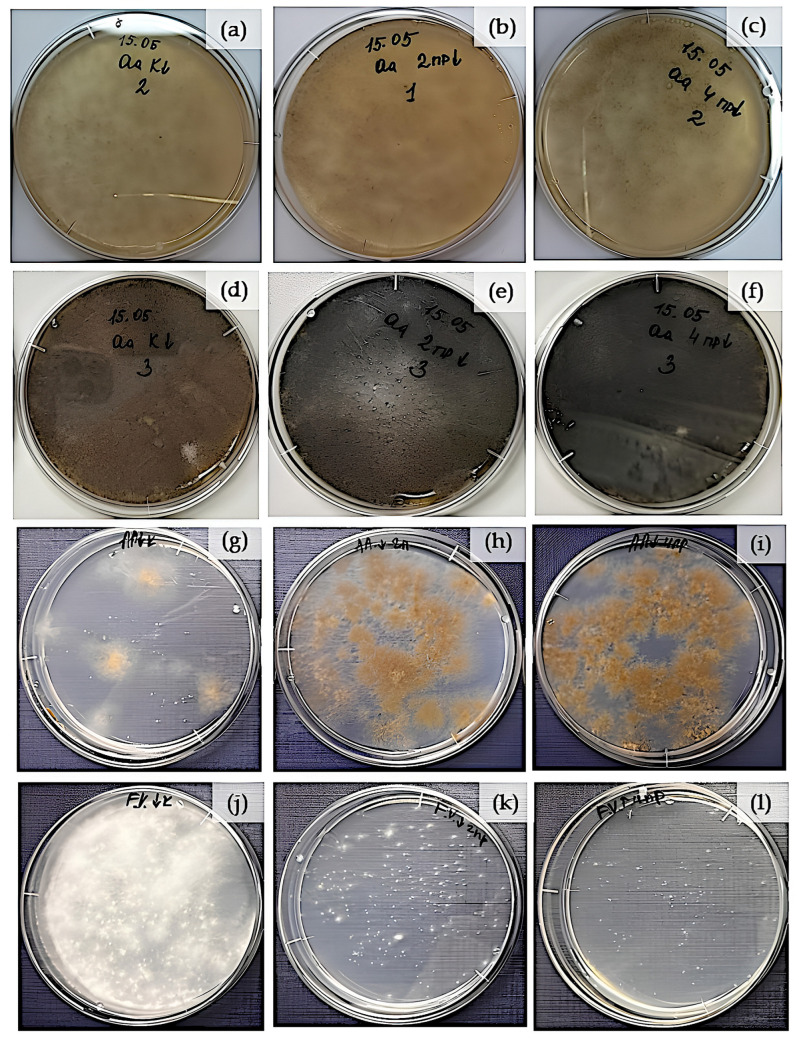
Morphological response of fungal cultures to sonoplasma treatment (SPT) under different experimental conditions. The figure illustrates the comparative growth of the hyaline fungus *F. solani* and the melanized fungus *A. alternata* after exposure to different SPT modes. Within each row, panels represent the control, double treatment, and fourfold treatment, respectively. (**a**–**c**): *A. alternata* colonies cultured from spore suspensions without storage after SPT, day 4 of growth, CFU assessment; (**d**–**f**): *A. alternata* colonies cultured from spore suspensions without storage after SPT, day 10 of growth, sporulation assessment; (**g**–**i**): *A. alternata* colonies cultured from spore suspensions stored for 10 days after SPT before inoculation, day 4 of growth, CFU assessment; (**j**–**l**): *F. solani* colonies cultured from spore suspensions stored for 10 days after SPT before inoculation, day 4 of growth, CFU assessment.

The hyaline fungus *F. solani* was more sensitive to direct SPT than *A. alternata* (Table 3). Treatment increased the time required for germination and reduced colony-forming recovery, as indicated by the shift from confluent lawn growth in the control to countable colonies after treatment. This reduction was observed at both initial spore densities (10^2^ and 10^4^ conidia/mL) and was stronger after fourfold treatment than after double treatment. Although colony-forming capacity decreased, conidial productivity increased in the surviving colonies: approximately 5.3- to 6.1-fold after double treatment and 3.6- to 4.0-fold after fourfold treatment relative to the untreated control (Figure 1j). The effect on mycelial biomass depended on both treatment intensity and initial spore density. At 10^2^ conidia/mL, biomass was significantly lower only after double treatment, whereas at 10^4^ conidia/mL, biomass decreased after both double and fourfold treatments.

**Figure 2:**
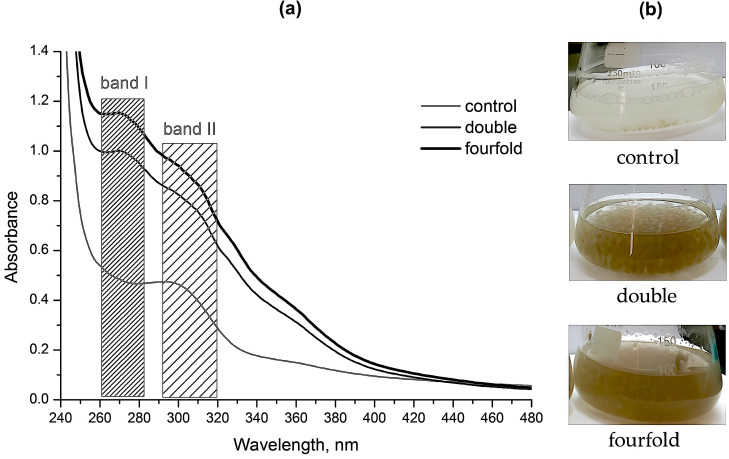
UV-visible absorption spectra of dissolved organic compounds in culture filtrates from *Alternaria alternata* following sonoplasma treatment (SPT) and untreated control (**a**); representative photographs of fungal pellets in the culture medium prior to filtration and spectral measurements (**b**).

The melanized fungus *A. alternata* showed a different response. Germination delay did not change, and lawn growth was maintained after both double and fourfold treatments (Table 3). Conidial productivity increased by approximately 1.4- to 1.5-fold relative to the untreated control. Mycelial biomass also increased after SPT, with treated cultures producing approximately 1.8- to 2.9-fold more biomass than the corresponding controls. Colonies on solid medium and pellets in liquid medium appeared darker after SPT than in the untreated control (Figure 1d–f and Figure 2b). This pigmentation response is described here as a phenotypic change; its possible relationship to melanization and stress tolerance is considered in the Discussion.

Quantitative data for immediate treatment effects are provided in Table 1 and Table 2.

### 3.3. Spectral Analysis of Dissolved Fungal Metabolites

Spectral measurements were used to characterize changes in dissolved organic compounds released during growth of *A. alternata* after SPT. The absorption spectra of the culture medium showed higher absorbance in the UV range (200–400 nm) in the double- and fourfold-treated variants than in the untreated control (Figure 2). This pattern was consistent with the higher mycelial biomass observed after SPT in Section 3.2.

The relative intensity of the absorption bands also differed among variants. The increase in band I, with a maximum near 270 nm and commonly associated with aromatic amino acids such as tyrosine and tryptophan or protein-like compounds, was more pronounced than the increase in band II, with a maximum at 305–310 nm and associated with other dissolved fungal metabolites [40]. These spectral differences indicate that SPT-treated cultures differed from the control in the composition or relative abundance of dissolved organic compounds. Because individual metabolites were not chemically identified in this experiment, these results should be interpreted as spectral indicators rather than direct evidence of specific metabolite production.

Excitation wavelengths of 270 nm and 310 nm were selected on the basis of the absorption bands identified in Figure 2. The 270 nm excitation corresponded to the absorption region of aromatic amino acids and protein-like substances, whereas the 305–310 nm region was associated with other dissolved organic compounds.

**Figure 3:**
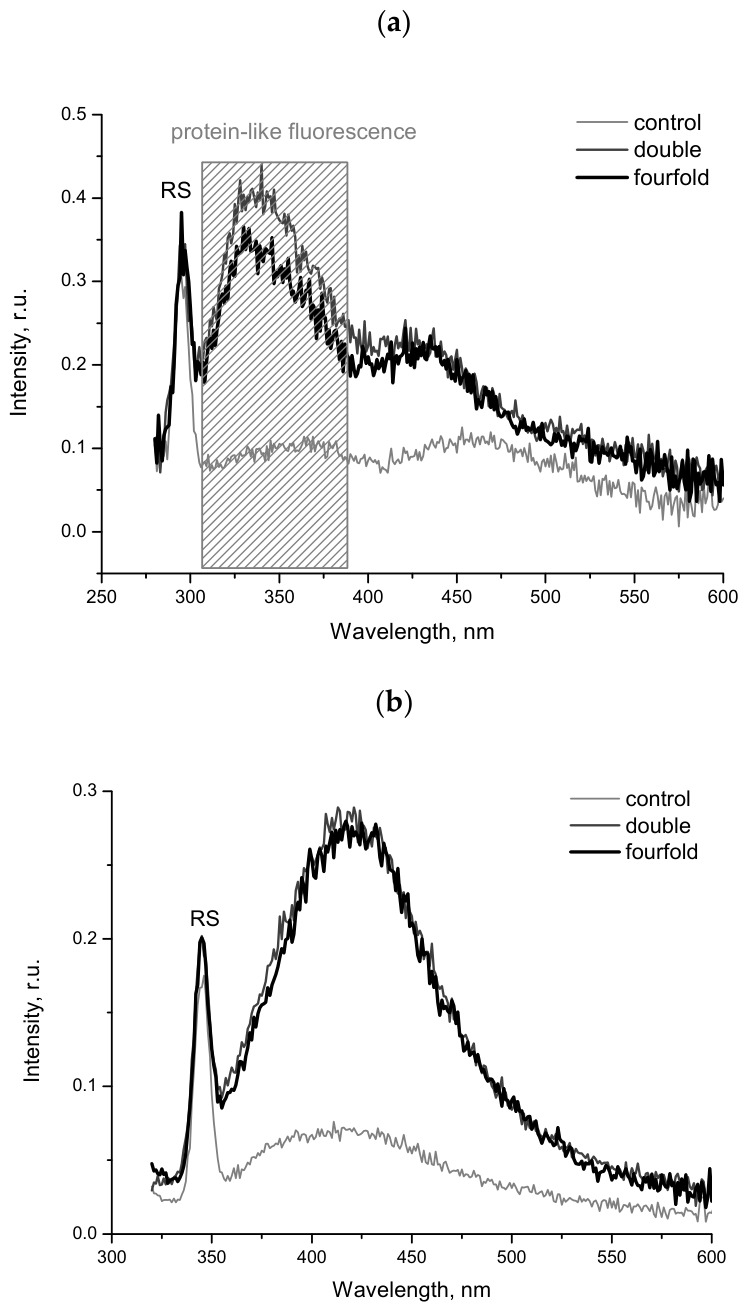
Fluorescence emission spectra of dissolved organic compounds in culture filtrates from *Alternaria alternata* following sonoplasma treatment (SPT) and in the untreated control. The figure compares the fluorescence intensity and spectral profiles of filtered (0.22-μm) culture filtrates following different SPT modes (control, double, and fourfold treatment). (**a**): emission spectra measured at an excitation wavelength of 270 nm, corresponding to protein-like substances. (**b**): emission spectra measured at an excitation wavelength of 310 nm, corresponding to other dissolved organic matter. RS indicates Raman scattering of light by water molecules.

Fluorescence spectra were consistent with the absorption data and showed higher emission intensity in SPT-treated *A. alternata* cultures than in the control (Figure 3a,b). A broad emission band in the blue region (400–500 nm), which may include contributions from melanin-like compounds and/or the cellular electron donor NAD(P)H, was more intense in treated samples (Figure 3b). This finding is consistent with the darker pigmentation observed in fungal pellets (Figure 2b), but it does not by itself identify the chemical origin of the signal.

The absorption and fluorescence spectra of the growth medium after cultivation of *A. alternata* in HP-containing medium did not show comparable increases in the spectral regions associated with protein-like or pigment-related compounds.

### 3.4. Allelopathic Activity of Fungal Cultures on Higher Plants

**Figure 4:**
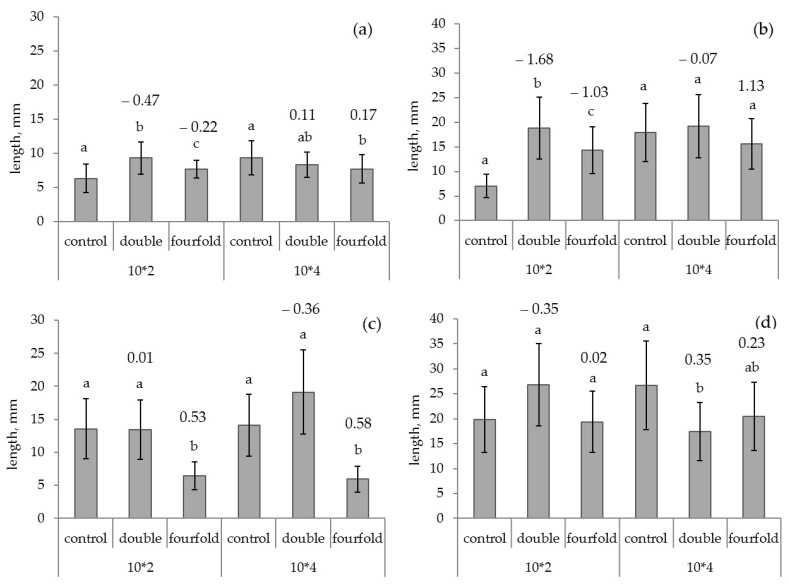
Effects of volatile or airborne metabolites from SPT-treated fungal cultures on *Avena sativa* seedlings. The figure presents the final measured lengths of roots (**a**,**c**) and coleoptiles (**b**,**d**) of common oat seedlings after 96 h of exposure to volatile or airborne metabolites from SPT-treated and untreated fungal cultures. Data are grouped by initial spore density (10^2^ and 10^4^ conidia/mL) for each species. (**a**,**b**): root length (**a**) and coleoptile length (**b**) in seedlings exposed to *F. solani* cultures. (**c**,**d**): root length (**c**) and coleoptile length (**d**) in seedlings exposed to *A. alternata* cultures. The values for allelopathic activity coefficients (Ca), calculated from inhibition of root and coleoptile length in *A. sativa* seedlings, are shown above the bars. Note: Variants sharing the same letter do not differ significantly (*p* ≥ 0.05). Comparisons were made among treatment modes within each species, initial spore density, and storage time.

SPT influenced the growth-inhibitory activity of fungal cultures toward common oat seedlings (*Avena sativa* L.) (Figure 4).

Overall, *A. alternata* showed stronger inhibitory effects on oat seedling growth than *F. solani*. The highest allelopathic activity coefficients (Ca > 0.5) were recorded for *A. alternata* variants exposed to the fourfold SPT mode. These results indicate that SPT-treated *A. alternata* cultures produced stronger inhibition of oat root and coleoptile growth in this assay. Possible changes in volatile-mediated growth-inhibitory activity are considered in the Discussion.

### 3.5. Fungal Growth Following Prolonged Storage After Sonoplasma Treatment

To determine whether the effects of SPT persisted after treatment, fungal growth parameters were analyzed after 10 and 30 days of refrigerated storage of the treated spore suspensions (Table 4).

Stored *F. solani* spore suspensions showed reduced culturability after SPT. Treated spores generally required longer to germinate than control spores, and CFU counts decreased after storage (Figure 1j–l). At the lower initial density (10^2^ conidia/mL), no colonies were recovered after fourfold treatment and 10 days of storage, and no colonies were recovered after either double or fourfold treatment after 30 days of storage. At the higher initial density (10^4^ conidia/mL), no colonies were recovered after fourfold treatment and 30 days of storage, whereas a low number of colonies remained after double treatment.

*A. alternata* showed a contrasting pattern (Table 4). CFU counts from SPT-treated spore suspensions were often higher than in the corresponding controls after storage, although the magnitude and statistical significance of this effect varied among treatment modes, storage times, and initial spore densities. At 10^2^ conidia/mL, both double and fourfold treatment increased CFU counts after 10 days of storage, and fourfold treatment remained higher than the control after 30 days. At 10^4^ conidia/mL, double treatment increased CFU counts after 10 days, and both double and fourfold treatment increased CFU counts after 30 days. These results indicate that the post-treatment storage response differed between the two fungi, with declining culturability in *F. solani* and retained or increased recovery in several *A. alternata* treatment variants.

### 3.6. Fungal Growth in Water After Sonoplasma Treatment

Exposure to SPT-treated water produced weaker effects than direct SPT exposure. No significant differences in biomass were observed between fungi exposed to SPT-treated water and the untreated-water control. CFU counts also did not differ significantly when *A. alternata* and *F. solani* were inoculated from water immediately after treatment or after storage (Table 5). Across treatment and storage variants, CFU counts ranged from 120.0 ± 15.9 to 192.0 ± 31.5 CFU/mL for *A. alternata* and from 257.0 ± 65.2 to 326.5 ± 18.6 CFU/mL for *F. solani*.

Conidial productivity showed a species-specific response. In *A. alternata*, the number of conidia produced by colonies inoculated from SPT-treated water did not differ significantly from the control, either immediately after treatment or after water storage (Table 5). In contrast, *F. solani* showed significantly increased conidial productivity when inoculated from SPT-treated water immediately after treatment and after 10 days of water storage. This effect was no longer significant after 30 days of storage.

These results indicate that SPT-treated water had a limited prolonged effect on fungal growth and development. Its main detectable effect was a transient increase in sporulation in the hyaline fungus *F. solani*, whereas no significant effect was observed for the melanized fungus *A. alternata* under the same conditions.

### 3.7. Fungal Growth Under Hydrogen Peroxide Exposure

HP affected the hyaline *F. solani* more strongly than the melanized *A. alternata* (Table 6). At 2 mmol/L, HP did not significantly alter *F. solani* biomass, CFU recovery, or conidial productivity compared with the control. At 6 mmol/L, HP strongly reduced *F. solani* biomass and CFU recovery. This reduction in colony-forming capacity was accompanied by a marked increase in conidial productivity, which was approximately 5.8-fold higher than in the control.

In *A. alternata*, neither 2 nor 6 mmol/L HP significantly affected mycelial biomass, CFU recovery, or conidial productivity under the tested conditions. These results indicate that the two fungi differed in their response to HP exposure, with greater susceptibility observed in *F. solani*.

## 4. Discussion

Sonoplasma treatment (SPT) exerted distinct effects on the two phytopathogenic fungi tested in this study. A pronounced inhibitory response was observed mainly for the hyaline fungus *F. solani* when SPT was applied directly to the spore suspension. In contrast, when *F. solani* was inoculated into SPT-activated water, the main detectable response was increased conidial productivity rather than reduced viability or biomass. The melanized fungus *A. alternata* showed a different pattern, maintaining viable growth under the tested conditions and displaying stimulation of several growth-related and reproductive endpoints even after direct SPT exposure. These results indicate that the biological outcome of SPT depends not only on treatment intensity but also on fungal species traits, potentially including pigmentation-associated stress tolerance.

SPT increased conidial productivity in both fungi. This response should be interpreted cautiously because enhanced sporulation does not necessarily indicate improved physiological status. In fungi, increased conidial production may occur as part of a stress response [41,42], reflecting the fact that conditions favoring sporulation often differ from those supporting maximal vegetative growth [43]. In the present experiment, reduced colony development of *F. solani* under direct SPT and HP exposure was accompanied by increased sporulation, suggesting a stress-associated shift from vegetative growth toward reproductive output (Table 7).

The stimulation observed in some treatment variants may be discussed in the context of hormesis, defined as a biphasic response in which low or sublethal levels of a stressor stimulate biological activity, whereas higher levels cause inhibition or death [32]. However, the mechanisms underlying this response were not directly resolved in the present study. SPT generates a complex stress environment involving dissociation of water molecules, ROS formation, shock waves, electromagnetic effect, and localized extreme temperatures. Therefore, the observed stimulatory responses should be regarded as treatment-associated physiological effects rather than evidence for a single causal pathway.

One measurable chemical outcome of SPT was the formation of HP. Reactive species generated by plasma discharge can drive oxidative transformation of chemical and biological components in water [22]. In this study, HP concentration increased in a treatment-dependent manner, reaching 2.70 ± 0.05 mmol/L after fourfold SPT, as shown in Table 2. HP can therefore be considered a key measurable oxidative effector formed during SPT. At the same time, HP alone cannot represent the full sonoplasma process because direct SPT also includes mechanical and electromagnetic stressors.

HP is a ubiquitous ROS that can damage cellular organelles and essential macromolecules, including nucleic acids, proteins, and lipids [44,45]. Because of its relatively long half-life compared with short-lived radicals and its ability to diffuse through membrane lipid bilayers, HP can alter membrane permeability and oxidize intracellular targets [46]. These properties explain its widespread use as an antimicrobial agent in medical and industrial sterilization, where it decomposes to water and oxygen with heat release [47]. However, resistant microorganisms may require relatively high inhibitory concentrations, and combined treatments are often more effective. For example, solar irradiation combined with HP can inactivate *F. solani* even under low solar irradiance [48], while HP combined with moderate heating, approximately 55–75 °C, can inactivate fungal propagules [5]. In our experiment, 6 mmol/L HP inhibited *F. solani* but did not suppress *A. alternata*, consistent with greater oxidative-stress tolerance in the melanized species.

Fungi possess robust defense systems against oxidative stress [49,50], including protection from ROS generated by host plants during defense responses [51]. For phytopathogenic fungi, ROS detoxification is essential for colonization and survival in host tissues [52]. This protection is mediated by multiple molecular, biochemical, and cellular mechanisms, and resistance to HP has been documented in fungi of the genera *Alternaria* and *Fusarium* [48,52]. The contrasting responses of *A. alternata* and *F. solani* in the present study are therefore consistent with species-specific differences in oxidative-stress management.

Melanin is an important component of fungal protection against oxidative stress [53]. In *A. alternata*, disruption of melanin biosynthesis can increase susceptibility to external stressors. For example, deletion of *Aabrm1*, which encodes scytalone dehydratase, increases sensitivity to oxidative stress [53]. *AaSet2* is likewise required for both melanin biosynthesis and oxidative-stress resistance [54]. Melanin production in *A. alternata* proceeds mainly through the DHN pathway, involving genes such as *pksA*, *brm2*, and the transcription factor *CmrA* [55,56]. This pathway is linked not only to pigmentation but also to morphogenesis, sporulation, and environmental stress tolerance. The darker pigmentation observed after SPT in *A. alternata* is compatible with activation of a protective or compensatory response. Nevertheless, because our study compared two natural species rather than melanin-deficient mutants or chemically inhibited melanization variants, the results should be interpreted as consistent with pigmentation-associated tolerance, not as direct proof that melanin alone caused the observed resistance.

The increased conidial productivity observed after exposure to activated water is also consistent with previous evidence that moderate HP exposure can induce hormetic responses. HP is a well-established hormetic trigger in *S. cerevisiae* [33]. Moderate intracellular HP can act as a signaling cue that activates defense systems [57], while superoxide radicals produced under HP stress may be protective at low concentrations but harmful at higher levels [58]. Catalase activity, which decomposes HP, is also central to yeast hormesis under HP exposure [33]. In the present study, discharge-activated water contained residual HP after activation and increased conidial productivity in *F. solani* in some indirect-treatment variants (Table 5). This is compatible with the possibility that residual oxidative activity in treated water can act as a sublethal signal under certain conditions, although the contribution of HP cannot be separated fully from other SPT-induced changes in water chemistry.

Mechanical effects are also likely to contribute to the biological response to direct SPT. Shock waves can inactivate microorganisms by damaging cellular structures, disrupting membranes, or generating pressure gradients large enough to cause structural failure at the scale of microbial cells [15,16,59]. During SPT, rapid local heating can promote vapor-bubble formation and collapse, producing mechanical forces that injure microbial cells; adaptation to this type of physical damage is considered unlikely [16]. However, hormetic stimulation by shock waves has not, to our knowledge, been demonstrated in microbial systems. Shock waves are therefore more plausibly associated with cellular injury during direct SPT, whereas the stimulatory effects observed after indirect exposure to activated water are more likely related to persistent chemical changes in the treated medium.

Radiation-based treatments are widely recognized for their effectiveness in fungal inactivation and reduction in mycotoxin content [60,61]. Their efficacy depends on multiple factors, including the physiological state and taxonomic group of the target fungi, radiation dose. Among electromagnetic radiation types, ionizing radiation is generally the most effective in inactivating conidia and reducing mycotoxin levels. Nonionizing radiation generated during SPT is less effective at fungal inactivation and require high intensities to achieve substantial effects [61]. Among nonionizing approaches, UV irradiation remains the most commonly applied disinfection method [6,8,12,13,14,62]. Additional studies have demonstrated that hot air-assisted radiofrequency treatments reduce *Aspergillus flavus* and *Penicillium crustosum* contamination [63], while microwave treatments effectively reduce *A. flavus* and *A. parasiticus* [64].

Temperature effects are unlikely to explain the species contrast by themselves. Both strains were isolated from biotopes with similar temperature regimes and are mesophilic, with optimal growth between 22 and 28 °C. However, melanized fungi may be more tolerant of thermal stress. Elevated temperatures can stimulate melanin synthesis and partial protein degradation, generating substrates for tyrosinase-dependent melanoidin formation; high tyrosinase activity in intensely pigmented forms has been associated with increased thermal resistance [65]. Therefore, localized thermal effects generated during SPT may have interacted with pigmentation-associated stress responses in *A. alternata*.

Taken together, the results indicate that *A. alternata* was more tolerant of the combined stresses generated by SPT than *F. solani* (Table 3 and Table 4). The shift from inhibition in the hyaline *F. solani* to stimulation in the melanized *A. alternata* is consistent with the hypothesis that pigmentation-associated traits can buffer oxidative and physical stress. However, other interspecific differences, including antioxidant enzyme activity, membrane composition, recovery capacity, and storage tolerance, may also have contributed. The increased allelopathic activity of *A. alternata* after fourfold SPT (Figure 4) further suggests that SPT-induced stress may alter not only growth and sporulation but also volatile-mediated growth-inhibitory activity. This finding is important for water-treatment applications because survival of resistant fungal propagules after treatment could be accompanied by altered physiological activity. However, whether survival of SPT-treated propagules is accompanied by retention or alteration of pathogenicity toward plant hosts was not tested and should be directly assessed in future work using appropriate inoculation assays.

Overall, this two-species model shows that SPT efficacy against filamentous fungi is species-dependent and may be limited when resistant melanized taxa are present. The findings support the need to optimize SPT parameters and to evaluate target microbial communities before practical application. Future studies should include broader fungal panels, melanin-deficient or melanin-inhibited strains, direct melanin quantification, expression analysis of melanization and oxidative-stress genes, HP dose–response and concentration-time response assays, pass-equivalent HP controls in pure water, and validation in natural waters or wastewater. These experiments will be needed to distinguish the relative roles of chemical, mechanical, electromagnetic, and thermal components of SPT and to determine whether the pigmentation-associated tolerance observed here applies more broadly across filamentous fungi.

## 5. Conclusions

Sonoplasma treatment produced species-specific effects on two phytopathogenic fungi with contrasting pigmentation phenotypes. Direct SPT reduced viability and biomass production in the hyaline *F. solani*, whereas the melanized *A. alternata* maintained viable growth under the tested conditions and showed stimulation of growth-related and reproductive endpoints. Both species showed increased sporulation after treatment, indicating that conidial production should be interpreted as a stress-associated response rather than as a simple measure of treatment success.

The observed contrast between *F. solani* and *A. alternata* is consistent with pigmentation-associated tolerance to SPT-induced oxidative and physical stress. However, because the study was based on two natural species and did not include melanin-deficient mutants, direct melanin measurements, or molecular validation, the results do not establish melanin as the sole causal mechanism. The HP experiment is consistent with a role of oxidative stress in the fungal response, but it did not isolate HP effects under conditions fully equivalent to direct SPT and does not reproduce the combined physical and chemical conditions generated during sonoplasma treatment.

The results also show that direct and indirect exposure to SPT can lead to different biological outcomes. Direct treatment was more effective against the susceptible hyaline fungus, whereas activated water alone increased conidial productivity in *F. solani* under some conditions. This distinction is important for practical applications because microorganisms in real water-treatment systems may be exposed either during discharge or after treatment, and these scenarios may not produce equivalent antifungal effects.

These findings indicate that SPT efficacy against filamentous fungi may be limited when resistant melanized taxa are present. Practical application of SPT for fungal control in water-treatment systems will therefore require optimization of treatment intensity, exposure duration, and repeated-treatment regimes. Future validation should be performed in mixed microbial communities and environmentally realistic waters, where organic matter, suspended particles, and microbial interactions may alter ROS persistence and treatment efficacy. Future studies should also determine whether surviving fungal propagules retain or alter their pathogenicity toward plant hosts after treatment. Broader fungal panels and mechanistic assays will be needed to determine whether the pigmentation-associated tolerance observed here applies more generally across filamentous fungi.

## Figures and Tables

**Table 1:** Experimental treatment design.

Treatment Options	Duration	*A. alternata*	*F. solani*
Direct effect of SPT	Immediate effect	+	+
	Prolonged effect	+	+
Indirect effect of SPT	Immediate effect	+	+
	Prolonged effect	+	+
Effect of HP	Immediate effect	+	+

“+” means experiments were carried out and data was obtained.

**Table 2:** Chemical parameters of the aquatic system following sonoplasma treatment (SPT). Values represent means ± standard deviation.

Treatment Mode	pH	H_2_O_2_, mmol/L
Control	7.92 ± 0.05	0.10 ± 0.05
Double	8.03 ± 0.05	1.30 ± 0.05
Fourfold	8.07 ± 0.05	2.70 ± 0.05

**Table 3:** Growth endpoints of fungal spore suspensions inoculated immediately after sonoplasma treatment (SPT). The table summarizes species-specific responses of *F. solani* and *A. alternata* to different SPT modes (control, double treatment, and fourfold treatment), assessed by germination delay, CFU recovery, mycelial biomass, and conidial productivity.

Species	Initial Spore Density, (Conidia/mL)	SPT Mode	Colony Growth		Mycelial Biomass, Dry Weight (g/100 mL)	Conidial Productivity (10^3^, Conidia/cm^2^)
			Germination Delay (Days)	CFU/mL		
*F. solani*	10^2^	Control	2	Entire lawn growth	0.122 ± 0.006 ^a^	293.7 ± 12.2 ^a^
	10^2^	Double	3	104.0 ± 14.5 ^a^	0.090 ± 0.004 ^b^	1784. 8 ± 81.7 ^b^
	10^2^	Fourfold	3	23.3 ± 3.2 ^b^	0.106 ± 0.005 ^ab^	1173. 2 ± 51.2 ^c^
	10^4^	Control	2	Entire lawn growth	0.109 ± 0.009 ^a^	343.7 ± 17.2 ^a^
	10^4^	Double	3	168.7 ± 11.0 ^a^	0.078 ± 0.004 ^b^	1834.8 ± 91.7 ^b^
	10^4^	Fourfold	3	63.0 ± 7.5 ^b^	0.061 ± 0.003 ^b^	1223.2 ± 61.2 ^c^
*A. alternata*	10^2^	Control	3	Entire lawn growth	0.040 ± 0.008 ^a^	125.2 ± 12.3 ^a^
	10^2^	Double	3	Entire lawn growth	0.109 ± 0.005 ^b^	186.3 ± 27.1 ^b^
	10^2^	Fourfold	3	Entire lawn growth	0.114 ± 0.006 ^b^	183.5 ± 28.8 ^b^
	10^4^	Control	3	Entire lawn growth	0.046 ± 0.002 ^a^	122.3 ± 8.2 ^a^
	10^4^	Double	3	Entire lawn growth	0.101 ± 0.005 ^b^	186.4 ± 33.0 ^b^
	10^4^	Fourfold	3	Entire lawn growth	0.084 ± 0.020 ^b^	166.0 ± 27.1 ^b^

Note: Variants sharing the same letter do not differ significantly (*p* ≥ 0.05). Comparisons were made among treatment modes within each species and initial spore density.

**Table 4:** Long-term viability and growth of fungal spores following SPT, assessed after 10 and 30 days of storage.

Species	Initial Spore Density, (Conidia/mL)	SPT Mode	10-Day Storage		30-Day Storage	
			Germination Delay (Days)	CFU/mL	Germination Delay (Days)	CFU/mL
*F. solani*	10^2^	Control	2	Entire lawn growth	2	129.0 ± 8.7 ^a^
	10^2^	Double	3	152.3 ± 44.8 ^a^	3	0 ^b^
	10^2^	Fourfold	3	0 ^b^	-	0 ^b^
	10^4^	Control	2	Entire lawn growth	2	73.3 ± 14.0 ^a^
	10^4^	Double	3	171.7 ± 31.9 ^a^	4	11.7 ± 2.5 ^b^
	10^4^	Fourfold	3	104.7 ± 5.0 ^a^	-	0 ^b^
*A. alternata*	10^2^	Control	4	9.0 ± 1.0 ^a^	4	5.3 ± 0.9 ^a^
	10^2^	Double	4	52.0 ± 6.9 ^b^	4	15.7 ± 3.7 ^a^
	10^2^	Fourfold	4	72.3 ± 18.8 ^b^	4	39.0 ± 9.5 ^b^
	10^4^	Control	4	12.0 ± 1.0 ^b^	4	5.3 ± 0.9 ^a^
	10^4^	Double	4	59.3 ± 17.0 ^a^	3	44.7 ± 14.0 ^b^
	10^4^	Fourfold	4	31.0 ± 7.9 ^b^	3	74.3 ± 9.1 ^c^

Note: Variants sharing the same letter do not differ significantly (*p* ≥ 0.05). Comparisons were made among SPT modes (control, double, fourfold) within each species and initial spore density, and storage time.

**Table 5:** Viability and conidial productivity of fungi following exposure to SPT-treated water, assessed immediately after treatment and after 10 and 30 days of water storage (initial spore density: 10^4^ conidia/mL).

Species	Conidial Productivity (10^3^ Conidia/cm^2^)			
	SPT Mode	Day 0	10-Day Storage	30-Day Storage
*F. solani*	control	3079.9 ± 226.5 ^a^	1797.3 ± 196.6 ^a^	2009.6 ± 803.2 ^a^
	fourfold	5821.2 ± 3602.7 ^b^	3376.1 ± 135.8 ^b^	2249.8 ± 401.6 ^a^
	Colonies grown from an aliquot of spore suspension (CFU/mL)			
	SPT mode	Day 0	10-day storage	30-day storage
	control	288.0 ± 19.4 ^a^	295.0 ± 54.5 ^a^	326.5 ± 18.6 ^a^
	fourfold	257.0 ± 65.2 ^a^	306.5 ± 27.0 ^a^	323.0 ± 80.8 ^a^
*A. alternata*	Conidial productivity (10^3^ conidia/cm^2^)			
	SPT mode	0 day	10-day storage	30-day storage
	control	56.8 ± 2.1 ^a^	52.4 ± 25.1 ^a^	65.5 ± 18.5 ^a^
	fourfold	49.5 ± 12.4 ^a^	76.2 ± 8.4 ^a^	24.8 ± 6.2 ^a^
	Colonies grown from an aliquot of spore suspension (CFU/mL)			
	SPT mode	0 day	10-day storage	30-day storage
	control	192.0 ± 31.5 ^a^	120.0 ± 15.9 ^a^	122.0 ± 16.3 ^a^
	fourfold	138.0 ± 34.0 ^a^	122.5 ± 15.5 ^a^	122.5 ± 11.9 ^a^

Note: Variants sharing the same letter do not differ significantly (*p* ≥ 0.05). Comparisons were made between control water and fourfold SPT-treated water within each species and storage time.

**Table 6:** Viability, conidial productivity of fungal cultures following exposure to hydrogen peroxide.

Species	Mode	Mycelial Biomass, Dry Weight (g/100 mL)	Conidial Productivity (10^3^ Conidia/cm^2^)	CFU/mL
*F. solani*	control	0.300 ± 0.071 ^a^	34,501.06 ^a^ ± 10,496.14	Entire lawn growth
	HP, 2 mmol/L	0.343 ± 0.016 ^a^	26,981.60^a^ ± 8995.39	Entire lawn growth
	HP, 6 mmol/L	0.010 ± 0.004 ^b^	199,339.47^b^ ± 43,060.68	7.67 ± 3.21
*A. alternata*	control	0.465 ± 0.055 ^a^	33,026.66^a^ ± 11,251.94	17.40 ± 2.16 ^a^
	HP, 2 mmol/L	0.470 ± 0.022 ^a^	60,942.05^a^ ± 19,656.09	29.83 ± 3.87 ^a^
	HP, 6 mmol/L	0.425 ± 0.146 ^a^	21,231.42^a^ ± 6349.08	30.00 ± 4.58 ^a^

Note: Variants sharing the same letter do not differ significantly (*p* ≥ 0.05).

**Table 7:** Summary of vegetative development and reproductive output in *A. alternata* and *F. solani* under direct SPT, indirect SPT, and HP exposure.

Treatment Options	Duration	*A. alternata*		*F. solani*	
		Colony Growth	Conidial Productivity	Colony Growth	Conidial Productivity
Direct effect of SPT	Immediate effect				
	Prolonged effect		ND		ND
Indirect effect of SPT	Immediate effect				
	Prolonged effect				
Effect of HP	Immediate effect				

Dark gray, inhibition; light gray, stimulation; white, no effect; ND, no data.

## Data Availability

The data presented in this study are available upon request from the corresponding author.

## Abbreviations

The following abbreviations are used in this manuscript:

ANOVA	Analysis of variance
CFU	Colony forming unit
HP	Hydrogen peroxide
ROS	Reactive oxygen species
SPT	Sonoplasma treatment
